# Risk factors for revision surgery in operative treatment of traumatic injuries of the olecranon and prepatellar bursa

**DOI:** 10.1186/s12891-022-05980-9

**Published:** 2022-11-23

**Authors:** T. Schöbel, G. Hantusch, R. Hennings, S. Schleifenbaum, C. Kleber, U. Spiegl

**Affiliations:** 1 Department of Orthopedic, Trauma and Plastic Surgery, University of Leipzig, Liebigstraße 20, D-04103 Leipzig, Germany; 2 ZESBO – Zentrum zur Erforschung der Stuetz- und Bewegungsorgane, Semmelweisstrasse 14, D-04103 Leipzig, Germany; 3 Fraunhofer Institute for Machine Tools and Forming Technology, Nöthnitzer Straße 44, D-01187 Dresden, Germany

**Keywords:** Bursa, Laceration, Olecranon, Prepatellar, Complication, Revision

## Abstract

**Introduction:**

Traumatic lacerations of the prepatellar (PB) and olecranon bursa (OB) are common injuries. The aim of this study was to gain descriptive data and to identify risk factors associated with complications that made revision surgery after primary bursectomy necessary.

**Material and methods:**

In this retrospective monocentric study at a level I trauma center, all patients with traumatic lacerations of the PB or OB who were treated with primary surgical bursectomy from 2015 to 2020 were analyzed.

**Results:**

150 consecutive patients were included. In 44% of cases, the PB was affected (*n* = 66), in 56% the OB (*n* = 84). The reoperation rate after surgical bursectomy was 10.7% (*n* = 16). The main cause of reoperation was wound infection (50%; *n* = 8). The most common pathogen of postoperative infections was Staphylococcus aureus (87.5%). Several comorbidities have been identified as risk factors for reoperation after primary surgical bursectomy, such as heart diseases, arterial hypertension, the use of antihypertensives and anticoagulation. In contrast, surgical expertise, use of drains, postoperative immobilization, and postoperative antibiotics had no statistically significant effect. A significantly higher postoperative infection rate (17.6%) was observed in patients who were operated more than 48 h after initial trauma.

**Conclusions:**

Given the limited recommendations for therapy of these common injuries, further investigations should focus on standardized therapeutic options for lacerations of the PB or OB. Delayed surgical interventions after trauma were associated with higher complication rates. Therefore, urgent surgery within 48 h after trauma may help to prevent revisions.

**Level of evidence:**

Level of evidence IV.

## Introduction

Traumatic lacerations of the prepatellar (PB) and olecranon bursa (OB) are common injuries due to the bursae’s exposition and superficial location [1]. Therefore, the PB and OB are the main location for septic bursitis [2, 3]. The most common pathogen for septic complications is *Staphylococcus aureus* [4]. With an incidence of 0.2% in trauma patients, the OB is reported to be affected more often (62.1%) than the PB (37.7%) [5]. There are no evidence-based recommendations for the management of traumatic bursitis [4]. Concepts for treatment vary significantly from open drainage or wound closure to surgical bursectomy or bursa reconstruction [1, 5]. Short-term adjuvant antibiotic therapy is recommended in severe infectious bursitis requiring hospitalization, though there is no statistically significant correlation between the total duration of antibiotic therapy and recovery[4]. Baumbach et al. evaluated current treatment concepts for acute lesions of the OB and PB in Germany, Austria, and Switzerland in 2012: in Germany, 85% of trauma or orthopedic surgeons performed a total bursectomy in case of traumatic bursa lesions [1], aiming to avoid an infection of the bursa, which may be promoted due to its lobular structure [3]. Immobilization was conducted in over 60% and antibiotic therapy in 45% of cases [1]. Recommendations for time between traumatic bursa lesions and therapy with surgical bursectomy vary from 12 to 72 h [6].

Generally, there is still a lack of epidemiologic data and evidence-based treatment strategies [1]. Hence, one aim was to gain descriptive data of these common injuries. The second aim of this study was to evaluate any risk factors that were associated with the need of revision surgery after surgical bursectomy of the OB or PB.

## Materials and methods

### Patients

This retrospective study was performed at a single level I trauma center. All patients with traumatic lacerations involving the PB or OB who were treated via surgical bursectomy from 2015 to 2020 were included in this study. Patients who refused operative treatment or had concomitant injuries of the affected region such as a fracture of the olecranon, the radial-head or the patella were excluded. The study was approved by the local ethics committee (vote-number 0083/21-ek). The investigation was performed according to the ethical standards of the institutional committee and the 1964 Helsinki declaration and its later amendments or comparable ethical standards [7]. Epidemiologic data (age, sex, site, height, body weight, body mass index, trauma mechanism and season of accident), risk factors (nicotine, drug and/or alcohol abuse, medical immunosuppression, anticoagulation and/or antihypertensives) and comorbidities (diabetes, peripheral arterial occlusive disease (PAOD), arterial hypertension, malignant neoplasms, hypothyroidism and any kind of heart disease (including coronary heart disease, cardiac arrhythmias, heart failure)) were collected retrospectively from the medical records.

### Surgical and postoperative management

All patients who suffered traumatic injuries of the knee or the elbow with lacerations of the PB or OB in clinical examination were admitted to the hospital and surgical bursectomy was recommended. During surgery, debridement of the wound margins and radical bursectomy through an open approach was performed in all patients. Differences in the operation and immediate postoperative management (use of a drainage, use/duration of systemic antibiosis, antibiotics used, type and duration of postoperative immobilization, time until discharge from hospital) and other peri- and intraoperative factors potentially influencing the outcome of the surgical bursectomy (time interval between admission and surgery, operation time, time of the day the operation was performed, professional expertise of the main surgeon, anesthetic procedure) were evaluated by scanning the operative protocols. In case swabs were taken at initial surgery, the results were evaluated. Nighttime was defined as 11:00 pm to 5:59am, daytime was defined as 6:00am to 10:59 pm. To investigate the urgency of surgical care, patients were categorized into 3 groups according to the time interval between accident and operation: within 24 h, between 24 and 48 h, more than 48 h after initial trauma.

### Outcome parameters

The patients were followed for the time of the in-hospital stay. The length of the in-hospital stay, complications and the need for revision surgeries were analyzed. Revision surgeries were defined as any unplanned surgery during the in-patient stay after initial treatment or after readmission to the hospital within two postoperative months. The rate of operative revision was our primary outcome. Thus, the study cohort was divided into two groups: (1) patients who needed to undergo a surgical revision after primary surgical treatment and (2) patients who did not need revision surgery or were not readmitted to the trauma center after primary surgical treatment. Wound infections are defined as postoperative infections with a positive pathogen detection. Contaminations were defined as positive pathogen detection in the initial swabs without clinical inflammation. Postoperative oedema without germ detection were defined as hematoma.

### Statistical analysis

The data were compared descriptively using Excel 2013 (Microsoft Corporation, Redmond, WA, USA). Furthermore, the data were examined statistically using SPSS 25.0 (SPSS®, Inc. Chicago, USA). The level of statistical significance was set at *p* < 0.05. Normal distribution was tested using the Shapiro–Wilk test. Non-normally distributed parameters were analyzed for statistical differences using the Mann–Whitney-U-Test. Most of the data were dichotomously (binary) nominally scaled and tested for correlation using the Phi- and Cramer-V test. Categorical variables were compared using Pearson's chi-squared test. Metric variables were analyzed using binary logistic regression. Odds ratios (ORs) were represented by error bar diagrams. Figures were created using Excel (Microsoft Corporation, USA).

## Results

### Descriptive data

One hundred fifty consecutive patients (mean age = 48.3 ± 21.3 years) with traumatic lacerations of the PB and OB were treated operatively by bursectomy. In 44% of cases, the PB was affected (*n* = 66), in 56% the OB (*n* = 84). Epidemiologic data of the study cohort comparing cases with need of revision (group 1) and those without (group 2) is presented in Table 1.

**Table 1:** Epidemiologic Data of the study cohort

	**Revision (*n* = 16)**		**No Revision (*n* = 134)**		***p***
	**Median**	**IQR**	**Median**	**IQR**	
**Mean Age in years**	54.0	(± 33.75)	44.0	(± 32.50)	0.652
**Body Mass Index in kg/m**^**2**^	26.0	(± 3.5)	24.0	(± 6.0)	0.204
**Male**	10 (62.5%)		80 (59.7%)		0.829
**Olecranon bursa**	7 (43.7%) right: 3 (42.9%)		77 (57.5%) right: 43 (55.8%)		0.296 0.521
**Prepatellar bursa**	9 (56.3%) right: 5 (55.6%)		57 (42.5%) right: 27 (47.4%)		0.296 0.521

**Fig. 1:**
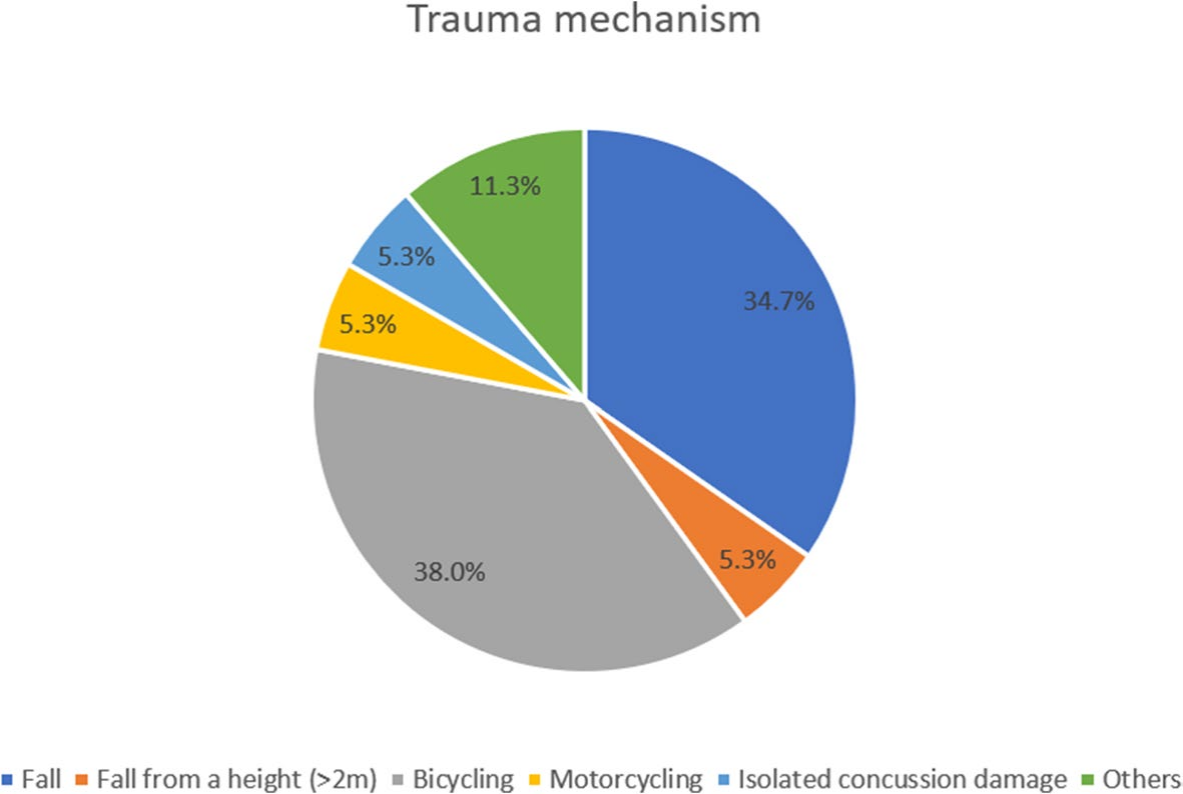
Trauma mechanism: Assaults, cuts, tool injuries, bite injuries, and high-velocity trauma were summarized as ''Others''

The main trauma mechanism is presented in Table 2 and Fig. 1.

**Table 2:** Main trauma mechanism

	**Revision (*n* = 16)**	**No Revision (*n* = 134)**	***p***
Fall	6 (37.5%)	46 (34.3%)	0.801
Fall from a height (> 2 m)	0 (0%)	8 (6.0%)	0.315
Bicycling	7 (43.8%)	50 (37.3%)	0.616
Motorcycling	1 (6.3%)	7 (5.2%)	0.863
Isolated concussion damage	1 (6.3%)	7 (5.2%)	0.863
Others	1 (6.3%)	16 (11.9%)	0.497

There was no statistically significant difference for age, sex, BMI, anatomical location or trauma mechanism between both groups.

**Fig. 2:**
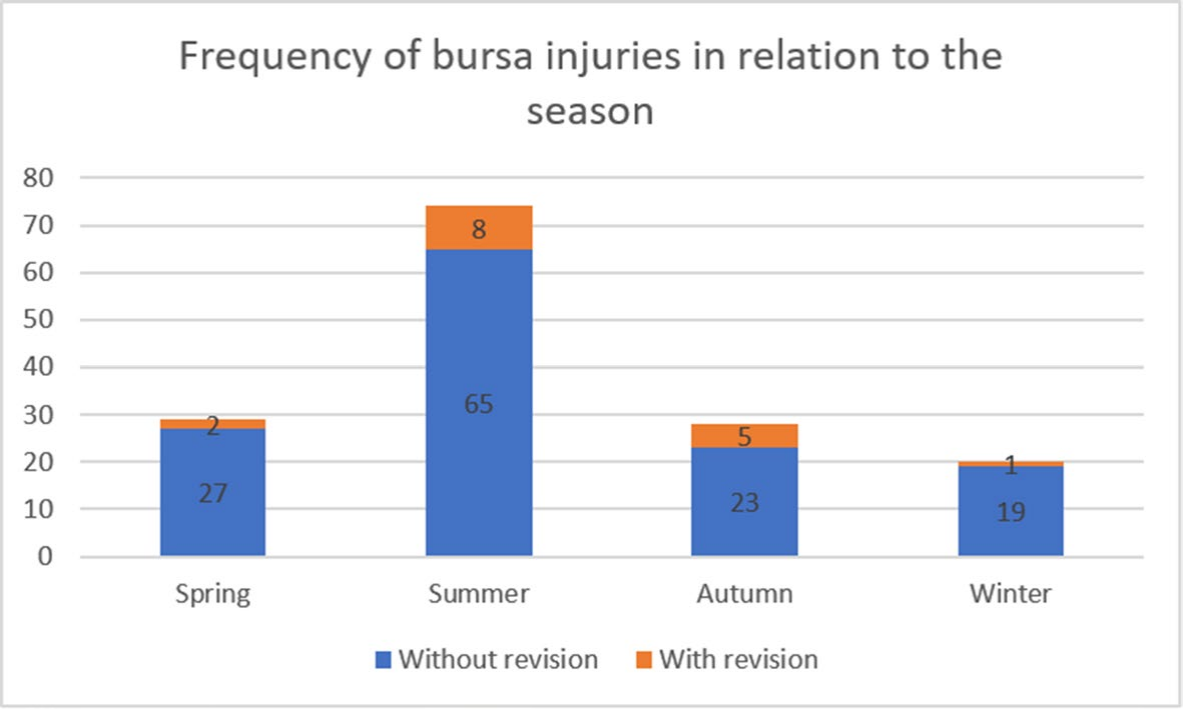
Frequency of bursa injuries in relation to the season

An accumulation of bursa injuries was observed in summer (June, July, August) (48.7%, *n* = 73). In contrast, the fewest bursa injuries were recorded in winter (December, January, February) (13.3%, *n* = 20) (Fig. 2).

Risk factors are presented in Table 3.

**Table 3:** Risk factors for revision after surgical bursectomy. Nominally scaled variables were tested for correlation using the Phi- and Cramer-V test. Categorical variables were compared using Pearson's chi-squared test

	**Total (*n* = 150)**	**Revision (*n* = 16)**	**No Revision (*n* = 134)**	***p***	**OR**	**95% CI**
Nicotine abuse	27 (18.0%)	4 (25.0%)	23 (17.2%)	0.441	1.61	0.48–5.44
Alcohol abuse	28 (18.7%)	4 (25.0%)	24 (17.9%)	0.492	1.53	0.45–5.15
Drug abuse	14 (9.3%)	2 (12.5%)	12 (9.0%)	0.645	1.45	0.29–5.15
Medical immunosuppression	11 (7.3%)	2 (12.5%)	9 (6.7%)	0.402	1.98	0.39–10.11
Anticoagulation	21 (14.0%)	5 (31.3%)	16 (11.9%)	**0.035**	**3.35**	1.03–10.9
Antihypertensives	38 (25.3%)	9 (56.3%)	29 (21.6%)	**0.003**	**4.66**	1.6–13.57
Diabetes	13 (8.7%)	3 (18.8%)	10 (7.5%)	0.129	2.86	0.7–11.73
PAOD	3 (2.0%)	0 (0.0%)	3 (2.2%)	0.546	-	-
Arterial hypertension	41 (27.3%)	9 (56.3%)	32 (23.9%)	**0.006**	**4.10**	1.41–11.88
Malignant neoplasm	9 (6.0%)	1 (6.3%)	8 (6.0%)	0.964	1.05	0.12–8.98
Heart disease	14 (9.3%)	6 (37.5%)	8 (6.0%)	**< 0.001**	**9.45**	2.74–32.66
Hypothyroidism	12 (8.0%)	3 (18.8%)	9 (6.7%)	0.094	3.21	0.77–13.34
Two or more comorbidities	22 (14.7%)	9 (56.3%)	13 (9.7%)	**< 0.001**	11.97	3.82–37.47
Only one comorbidity	36 (24.0%)	0 (0.0%)	36 (26.9%)	**0.0174**	-	-
No comorbidities	92 (61.3%)	7 (43.8%)	85 (63.4%)	0.127	0.45	0.16–1.28

### Reoperations

**Fig. 3:**
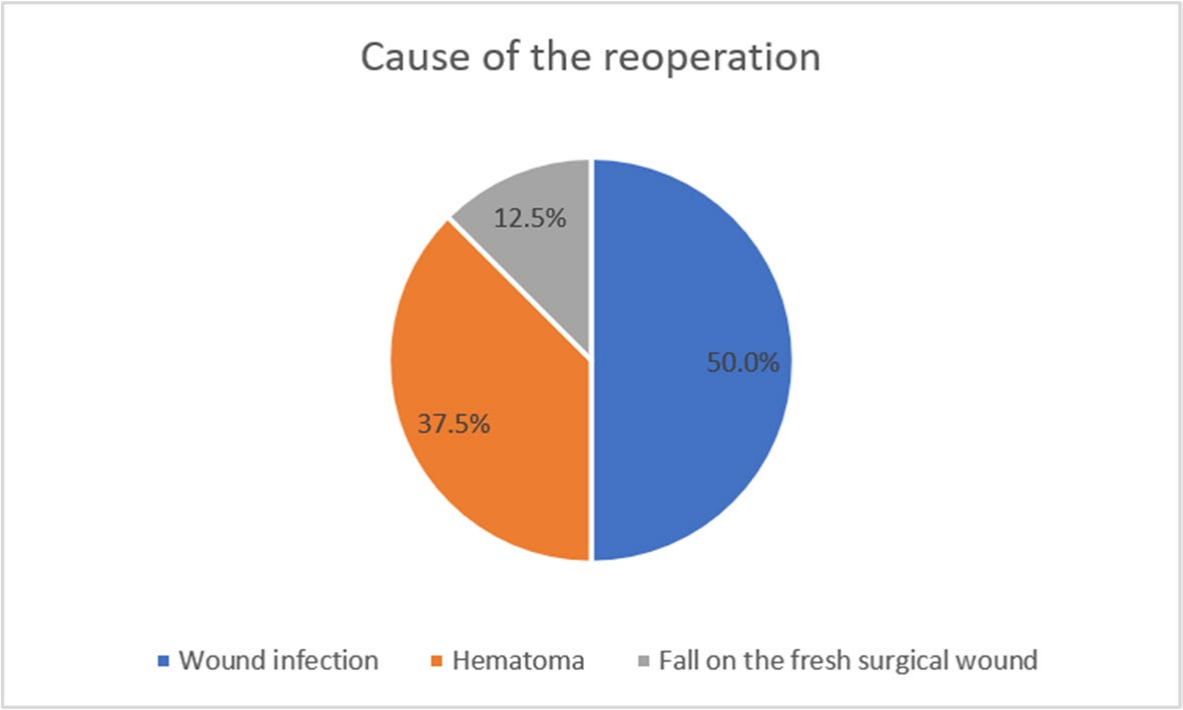
Cause of reoperation after surgical bursectomy

The rate of revision after surgical bursectomy was 10.7% (*n* = 16). The main cause of reoperation was wound infection in 50.0% (*n* = 8) of the cases. If there was a visible inflammation of the wound, a reoperation was indicated. The second most common cause was increased hematoma formation requiring evacuation (37.5%, *n* = 6). The remaining 12.5% (*n* = 2) were reoperated due to another fall on the fresh surgical wound with subsequent wound dehiscence and infection (Fig. 3). Among those patients who had an infection requiring revision surgery (*n* = 8), the majority (*n* = 7) were found to have *Staphylococcus aureus* on cultures (87.5%). The pathogens are presented in Table 4. Hematoma evacuations were significantly more likely to affect elderly patients, mean age 68.2 (Median = 74.0 years; IQR = 29.75) years (*p* = 0.023). Infection-related revisions, on the other hand, affected patients with a mean age of 39.4 (Median = 36.5 years; IQR = 29.50) years. The median time between trauma and revision surgery was 13.0 days (IQR = 23.0; range 1- 60 days).

**Table 4:** Identified pathogens of wound infection. Overall, swabs were obtained in 55.3% (*n* = 83) of cases

	**Infection (*n* = 8)**	**Contamination (*n* = 12)**
*Staphylococcus aureus*	7 (87,5%)	2 (16.7%)
*Streptococcus pyogenes*	1 (12.5%)	
*Staphylococcus epidermidis*		3 (25.0%)
*Staphylococcus capitis*		1 (8.3%)
*Staphylococcus haemolyticus*		1 (8.3%)
*Staphylococcus saphrolyticus*		1 (8.3%)
*Streptococcus dysgalactiae*		1 (8.3%)
*Acinetobacter baumanii*		1 (8.3%)
*Pantoea aglomerans*		1 (8.3%)
*Streptococcus viridans*		1 (8.3%)

### Risk factors for reoperations

**Fig. 4:**
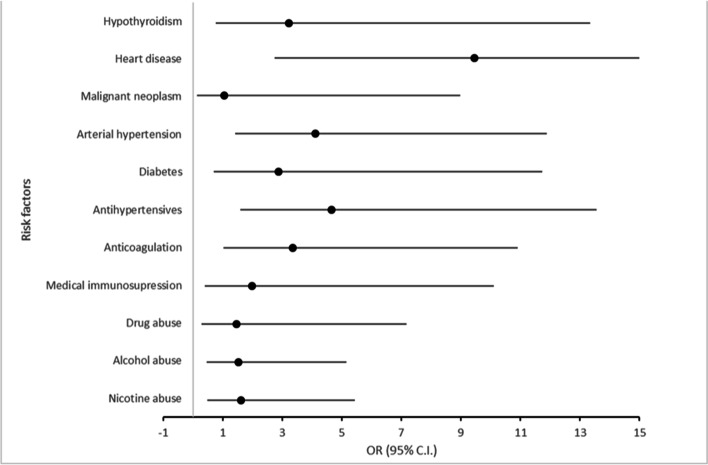
Error bar plot of risk factors for complications after surgical bursectomy

The groups with and without surgical revision after primary bursectomy differed statistically significant in terms of the number of comorbidities (*r* = 0.441; *p* < 0.001). Risk factors that led to a significantly increased rate of revision surgeries after primary surgical bursectomy were heart diseases (OR 9.45 (2.74–32.66); *r* = 0.335; *p* < 0.001), arterial hypertension (OR 4.1 (1.41–11.88); *r* = 0.224; *p* = 0.006), the use of antihypertensives (OR 4.66 (1.6–13.57); r 0.246; *p* = 0.003) and anticoagulation (OR 3.35 (1.03–10.9); *r* = 0.172; *p* = 0.035) (Fig. 4). Patients with two or more comorbidities were more likely to undergo revision surgery than patients with only one or no comorbidities (*p* < 0.001). No significant correlation with the occurrence of reoperations was found for the other risk factors shown in Table 3. However, the rate of postoperative wound infections (*n* = 8) was statistically significant higher in patients who were under immunosuppressive therapy (OR 4.93 (0.87–27.98); *r* = 0.161; *p* = 0.049) and in those who had hypothyroidism (OR 8.87 (1.82–43.16); *r* = 0.258; *p* = 0.002).

### Intraoperative and postoperative procedure

The primary surgery was performed within 24 h after the accident in 72.0% (*n* = 108) of patients, in 16.7% (*n* = 25) between 24 and 48 h and in 11.3% (*n* = 17) after 48 h, respectively. A significantly higher postoperative infection rate (17.6%) was observed in patients who were operated more than 48 h after initial trauma (*r* = 0.196; *p* = 0.016). With an increasing time interval between accident and surgery, there were significantly more germ detections in the swabs taken (*r* = 0.284; *p* = 0.001). Overall, germ swabs were obtained in 55.3% (*n* = 83) of cases. Among patients in group 1, 100% (*n* = 16) had a swab; among group 2 only 50% (*n* = 67). Of all intraoperative germ swabs 24.1% (*n* = 20) were positive. The most common pathogen was *Staphylococcus aureus* in 45% of all cases. There is a strong significant association between positive germ detection and revision (*r* = 0.5; *p* < 0.001). The most commonly used anesthetic procedure was balanced general anesthesia with a laryngeal mask (76.7%, *n* = 115). Bursectomies were performed under the supervision of a specialist (board-certified trauma surgeons) by residents in 67.3% (*n* = 101) of cases, in 19.3% (*n* = 29) by specialists, and in 13.3% (*n* = 20) by senior physicians, respectively. The median duration of surgery was 30 (IQR = 16.5) minutes. Senior physicians operated on average 7 min faster than residents. Specialists were in the middle range. Surgeon expertise (*p* = 0.348) and operative time (*p* = 0.904) had no significant association with the occurrence of revision surgery. Surgeries were performed at night in 52.0% (*n* = 79) of cases, and in 48.0% (*n* = 72) of cases during the day, without significant correlation in terms of increased rate of revision surgery (*p* = 0.374). A drain was placed in 60.7% (*n* = 91) of patients. This had no effect on outcome in terms of hematoma development (*p* = 0.570) or revision surgery (*p* = 0.675). A splint was applied for immobilization in 35.3% (*n* = 53) of patients. This did not reduce the rate of revision surgery (*p* = 0.865). Perioperative antibiosis was given to 75.3% (*n* = 113) of the patients. The patients without antibiosis did not have a significantly increased risk of revision surgery (*p* = 0.266).

## Discussion

Our first aim was to give a descriptive analysis of the trauma mechanisms and epidemiology of traumatic lacerations of the PB or OB. Our second aim was to identify risk factors and parameters in the treatment of patients who underwent surgical bursectomy after a traumatic lesion of the PB and OB that influenced the outcome of the procedure and consecutively made revision surgery necessary. Thereby, several risk factors could be identified, including heart diseases, arterial hypertension, immunosuppressive therapy, the use of antihypertensives, anticoagulation, and surgery after more than 48 h.

To our knowledge, there is only one published study that gave an epidemiologic description of patients with traumatic lacerations of the OB and PB: Raas et al. identified 552 patients with these traumata between 2009 and 2014 in a literature review [5]. Their data confirm most findings of the present study: the age of the patients was between 40 and 50 years, the OB was affected more often than the PB. They also found that most of the injuries occurred during summer [5]. This may be easily explained by the trauma mechanism: most of the lacerations were caused by activities that are more frequently done during warm months, like bicycling or activities such as jogging or football (which can lead to fall from low heights)and it is known that increased temperature is positively correlated with trauma admissions [8, 9]. Additionally, the protective effect of long shirts, sweaters or pans are less commonly seen in the summer months compared to the winter. The main trauma mechanism in their systematic review was a fall during daily activities (43.8%) [5]. In contrast, we observed mostly bicycle accidents (38%), which may be explained due to regional differences.

Rass et al. found complications for 19.8% of all observed lacerations of the OB or PB independent of the therapy (surgical or conservative). Infections or wound healing disturbances were found in 13% of patients [5]. We observed a lower complication rate of only 9.3% in our cohort, which might support the more aggressive strategy of a primary surgical resection of the lacerated bursa compared to primary conservative treatment [1]. Similarly, Raas et al. reported a lower complication rate in those patients who were treated operatively (19.1%) than in the group of patients who were treated conservatively (22.6%), despite the assumption, that in those patients the bursal affections might be more severe, what made the candidates for surgery [5].

Although alcohol, nicotine and drug abuse are known to induce delayed healing and wound complications [10–12], there was no statistically significant correlation between these risk factors and complications or the need revision surgery in the present study. This might be explained due to the fact that in the medical documentation the severity of alcohol, nicotine or drug abuse was not sufficiently documented (for example as “pack years”). Thus, no differentiation was possible between occasional users and people with addiction and regular abuse of these substances.

Our findings suggest that the number of comorbidities increased the risk for revision surgery after surgical bursectomy. Some studies have shown a correlation between age [5, 13] or diabetes [14, 15] in patients with skin lacerations and the likeliness to develop an infection [16]. The correlation between heart diseases, arterial hypertension, hypothyroidism and the use of antihypertensives and the risk for complications is not described in the recent literature and seems to be contradictory. On the other hand, the observed association of immunosuppressive medication and infections or wound healing disorders and of anticoagulation and hematoma is explainable due to the obvious side effects of these medications [17–19].

Regarding the time between accident and operative treatment, the dogma of wound closure until six to eight hours after accident is increasingly questioned. A multicenter study of 2663 patients who underwent treatment after lacerations in an emergency room found there was no statistically significant difference regarding wound infection between patients who were treated more than 12 h after injury and those who were treated within 12 h of the accident [15]. However, in the present study, there was a significantly higher infection rate in patients who were operated after more than 48 h after initial trauma. This may be explained due to a selection bias: patients with worsening wounds or deterioration of their general condition may have been more likely to see a surgeon than patients with similar trauma who did not experience complications. However, urgent surgery might help to reduce the revision rate. Expertise of the performing surgeon and the operative time did not statistically influence the revision rate.

Individual surgical decisions like the placement of a drain or postoperative immobilization (e.g. by using a splint) had no statistical effect on the rate of revisions in the present investigation. However, the duration of drain in situ and the duration of immobilization could not be evaluated retrospectively. Similarly, there might be a selection bias. Drains and splints might be dominantly used in cases with critical wound situations. A systematic review described a positive effect of cast immobilization and bandages leading to shorter treatment times [5]. Similarly, the usage of a drain was recommended and investigated in 50 patients with traumatic lesions of the PB in 2013 by Kaiser et al., resulting in a reduced revision rate of only 8% [6]. Thus, both drain and immobilization should be considered after surgical bursectomy.

Antibiotic therapy had no effect on the rate of revision surgery. Again, this finding has to be interpreted carefully due to a possible bias in the medical documentation and the retrospective test setting. Additionally, these results are contradictory to both our findings with a significant higher proportion of bacterial identification in patients of group 1 and the findings of Raas et al., who found that increasing antibiotic prophylaxis was associated with a reduction of the likelihood of infection [5]. On the other hand, Kaiser et al. only used antibiotic prophylaxis in patients who were immunosuppressed, polymorbid or showed deep and/or highly contaminated wounds [6]. This is supported by Moran et al., who stated that though most traumatic wounds do not require antibiotic prophylaxis, wounds in which the risk of infection is high enough justify prophylaxis. This includes immunocompromised patients, wounds into joints or involving tendons or cartilage and grossly contaminated wounds [20].

This is a retrospective study with some inherent flaws. (1) Though it allows explorative analyses, a possible selection bias should be considered. (2) Furthermore, patients who were treated initially and went for further treatment to other physicians or clinics may be not included. A standardized follow-up did not take place. This may influence the complication rate. (3) A possible affection of factors like trauma mechanism, concomitant injuries, size of the wound or comorbidities on clinical decision making can’t be ruled out and the findings of this study are highly dependent on the quality of the documentation. (4) The comorbidities were limited to diabetes, peripheral arterial disease, arterial hypertension, malignant neoplasms, hypothyroidism, and any type of heart disease. (5) No multivariate analysis or subgroup analysis is suitable based on the limited number of patients included.

## Conclusion

Traumatic lacerations of the PB or OB are common injuries, but there is a lack of epidemiologic data. Given the limited recommendations for therapy of these common injuries, further investigations should focus on standardized therapeutic options in case of lacerations of the PB or OB. Particularly in patients with comorbidities that were associated with a higher risk of revision surgery, risk factors need to be analyzed prospectively. This needs to be included in the informed consent prior to the surgery. Urgent surgery within 48 h after trauma helped to reduce the revision rate. Additionally, intraoperative swabs should be standardized to allow optimal antibiotic therapy in case of complications due to bacterial colonization.

## Data Availability

The datasets used and/or analyzed during the current study are available from the corresponding author on reasonable request.

## Abbreviations

BMI: Body mass index
IQR: Interquartile range
OB: Olecranon bursa
PB: Prepatellar bursa
CI: Confidence interval
OR: Odds ratio
SD: Standard deviation
